# Savannah

**Published:** 1858-11

**Authors:** 


SAVANNAH, Tenn., Oct. 7, 1858.

JNO. T. TOLAND, D. D. S.

*Dear Sir*:—For several years past my attention has been particularly directed to one of the most important branches connected with Dental Surgery, viz: The proper treatment of the dental pulp after exposure, and consequent inflammation.

It is an admitted fact, that all the remedies now used, are uncertain and precarious in their nature, and I confess promptly that my experience in such cases, has been far from satisfactory, although I have been experimenting for the last ten or twelve years.

It is my humble opinion that the dental faculty should have a standard, or at least a regular system of practice. As far as my observation extends, in a majority of instances, each operator has his own formula. All other branches of the art have advanced rapidly. This to some extent is an exception, and thousands of valuable teeth are lost annually, through ignorance or want of attention. I trust your highly popular and valuable *Reporter* will give us the best information extant, on this important subject.

Respectfully, SAVANNAH.

